# Evaluation of lateral pterygoid muscle in patients with temporomandibular joint anterior disk displacement using T1-weighted Dixon sequence: a retrospective study

**DOI:** 10.1186/s12891-022-05079-1

**Published:** 2022-02-08

**Authors:** Shuo Wang, Yu Chen, Dejun She, Zhen Xing, Wei Guo, Feng Wang, Hongjie Huang, Nan Huang, Dairong Cao

**Affiliations:** Department of Radiology, The First Affiliated Hospital of Fujian Medical University, 20 Cha-Zhong Road, Fuzhou, 350005 Fujian China

**Keywords:** Dixon sequence, Anterior disk displacement, Lateral pterygoid muscle, Fatty infiltration, Texture analysis

## Abstract

**Background:**

Pathological alterations of lateral pterygoid muscle (LPM) are implicated in temporomandibular joint anterior disk displacement (ADD). However, quantification of the fatty infiltration of LPM and its correlation with ADD have rarely been reported. The aim of this study was to evaluate the fatty infiltration, morphological features and texture features of LPM in patients with ADD using T1-weighted Dixon sequence.

**Methods:**

This retrospective study included patients who underwent temporomandibular joint MRI with T1-weighted Dixon sequence between December 2018 and August 2020. The temporomandibular joints of the included patients were divided into three groups according to the position of disk: Normal position disk (NP) group, Anterior disk displacement with reduction (ADDWR) group and Anterior disk displacement without reduction (ADDWOR) group. Fat fraction, morphological features (Length; Width; Thickness), and texture features (Angular second moment; Contrast; Correlation; Inverse different moment; Entropy) extracted from in-phase image of LPM were evaluated. One-way ANOVA, Welch’s ANOVA, Kruskal–Wallis test, Spearman and Pearson correlation analysis were performed. Intra-class correlation coefficient was used to evaluate the reproducibility.

**Results:**

A total of 53 patients with 106 temporomandibular joints were evaluated. Anterior disk displacement without reduction group showed higher fat fraction than normal position disk group (*P* = 0.024). Length of LPM was negatively correlated with fat fraction (*r* = -0.22, *P* = 0.026). Angular second moment (*ρ* = -0.32, *P* < 0.001), correlation (*ρ* = -0.28, *P* = 0.003) and inverse different moment (*ρ* = -0.27, *P* = 0.005) were negatively correlated with fat fraction, while positive correlation was found between entropy and fat fraction (*ρ* = 0.31, *P* = 0.001). The intra-class correlation coefficients for all values were ranged from 0.80 to 0.97.

**Conclusions:**

Patients with ADDWOR present more fatty infiltration in the LPM compared to NP or ADDWR patients. Fatty infiltration of LPM was associated with more atrophic and higher intramuscular heterogeneity in patients with ADD. Fat fraction of LPM quantitatively and noninvasively evaluated by Dixon sequence may has utility as an imaging-based marker of the structural severity of ADD disease process, which could be clinical helpful for the early diagnose of ADD and predication of disease progression.

## Background

Temporomandibular joint anterior disk displacement (ADD) is one of the most common subtypes of temporomandibular joint disorders (TMD), which is characterized by joint area and/or masticatory pain, joint movement disorders and joint noise [1, 2]. Lateral pterygoid muscle (LPM) attaches to the condylar head, capsule and possibly the disk [3]. Due to the tight connection between LPM and temporomandibular joint (TMJ), dysfunctional LPM such as hyperactivity and hypoactivity is considered to contribute the occurrence of ADD [4]. Several studies have shown that pathological alterations of LPM [4–6], especially the fatty infiltration [1], is closely related to ADD. Fatty infiltration could impair the muscle function and is accompanied by changes in muscle morphology, and this degradation is irreversible and might even worsen [7]. However, at present, the evaluation of fatty infiltration in LPM are all based on visual qualitative methods of conventional MRI [8–10]. These qualitative methods are easy to be subjectively affected, and can hardly detect the early minor fatty alteration of LPM. Quantitative MRI technique which can assess the muscle fatty infiltration could improve the insight in the degree of degeneration of LPM and might contribute to better ADD treatment strategy.

Dixon-based quantitative MRI technique can separate fat and water through the chemical shift analysis, allowing for direct fat quantification, and could detect subtle fatty alteration that cannot observed with the naked eye [11]. It has mainly used in limb to assess the muscle fatty infiltration in different conditions, including neuromuscular diseases [12], spinal muscular atrophy [13] and rotator cuff tears [14]. The head has rich tissue-air interfaces, which would cause magnetic field inhomogeneity and thus affecting the imaging quality in conventional MRI [15]. Owing to its short acquisition time and low sensitivity to susceptibility, Dixon-based method is very suitable for head muscle imaging and has been applied preliminary in recent years [15–17]. Latest literature showed that Dixon-based sequence presented better image quality for extraocular muscles [15]. Moreover, it was also found the fat fraction of extraocular muscles is higher even in early stage orbitopathy patients than that in healthy people by using Dixon technique [17]. Since there are abundant tissue-air interfaces around LPM as well as the extraocular muscles, these studies hinted that the Dixon-based technique also has the potential to be used in the assessment of LPM. However, Dixon-based technique was rarely reported in the evaluation of LPM up to now. Texture analysis can assess the grayscale patterns and pixels interrelationships in tissue that human eyes cannot distinguish, and can also quantitatively evaluate the heterogeneity of tissue [18, 19]. A considerable number of studies have confirmed that texture analysis can be used to quantitatively discriminate normal muscle from myopathic muscle [20–23]. What’s more, recent study showed that texture analysis of in-phase image of Dixon sequence might be used to identify the tissue with more heterogeneity [24]. Therefore, it can be speculated that the texture features extracted from in-phase image are also related to fat fraction of LPM considering the heterogeneity of muscle might be affected by fatty infiltration.

Thus, the purpose of this research was to quantify the fat fraction and morphological change of LPM with related to ADD, and to define whether fatty infiltration is correlated with intramuscular heterogeneity.

## Methods

### Patients

**Fig. 1:**
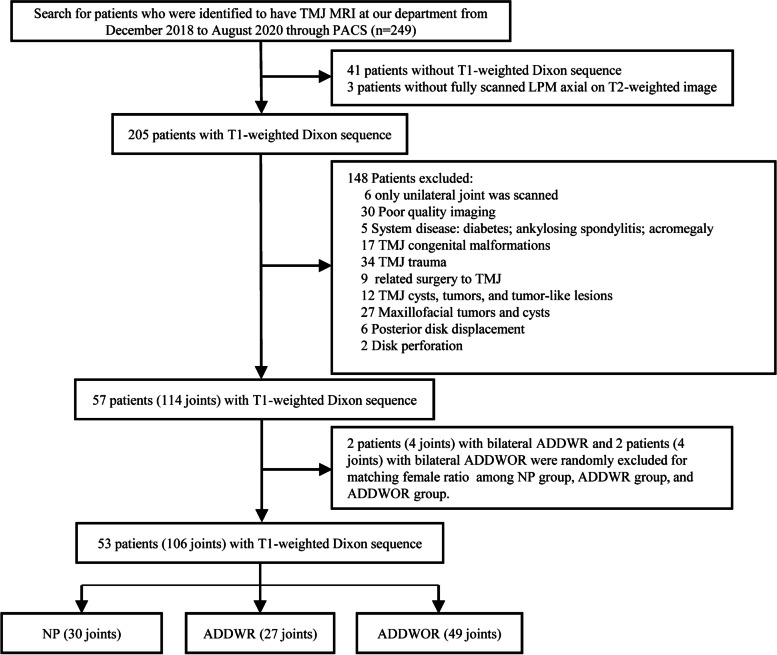
Flow chart of subject collection and exclusion criteria TMJ  Temporomandibular joint, PACS  Picture archiving and communication system, LPM, Lateral pterygoid muscle, NP, Normal position disk, ADDWR, Anterior disk displacement with reduction, ADDWOR, Anterior disk displacement without reduction

Patients were identified to undergo TMJ MRI at our department from December 2018 to August 2020. The exclusion criteria are as follows: (a) patients with MRI examination that without T1-weighted Dixon sequence, (b) patients without fully scanned LPM on the axial T2-weighted image, (c) only unilateral joint was scanned, (d) poor quality of MR images, (e) system disease: diabetes, ankylosing spondylitis and acromegaly, (f) TMJ congenital malformations, (g) TMJ trauma, (h) previous surgery related to TMJ, (i) TMJ cysts, tumors, and tumor-like lesions (j) maxillofacial tumors and cysts, (**k**) the posterior disk displacement as the small sample size (*n* = 6), (l) the disk perforation. A total of 57 patients with 114 joints were enrolled. The flowchart of collection and exclusion of patients is shown in Fig. 1.

### Data acquisition

All examinations were performed using 3.0-Tesla MRI scanner (Skyra, Siemens Healthcare, Erlangen, Germany) with 8 channel TMJ surface coil (MRCCA08SS30, TS-imaging, Beijing, China). The sagittal localizer image was first obtained, then the axial and coronal T2-weighted sequence were scanned. In the closed-mouth position, oblique sagittal proton density-weighted sequence with fat saturation, oblique sagittal T1-wighted sequence, oblique coronal proton density-weighted sequence with fat saturation, and coronal T1-weighted Dixon sequence were scanned. In the scanning of the coronal T1-weighted Dixon sequence, in-phase image and opposed-phase were obtained firstly, then the fat image and water image were automatically reconstructed by the post processing software of the scanner with the following formulas [25]:

$$\mathrm{Fat image}=\left({\mathrm{Signal Intensity}}_{\mathrm{In}-\mathrm{phase image}} - {\mathrm{Signal Intensity}}_{\mathrm{Opposed}-\mathrm{phase image}}\right)/2$$  

$$\mathrm{Water image}=\left({\mathrm{Signal Intensity}}_{\mathrm{In}-\mathrm{phase image}} + {\mathrm{Signal Intensity}}_{\mathrm{Opposed}-\mathrm{phase image}}\right)/2$$

**Fig. 2:**
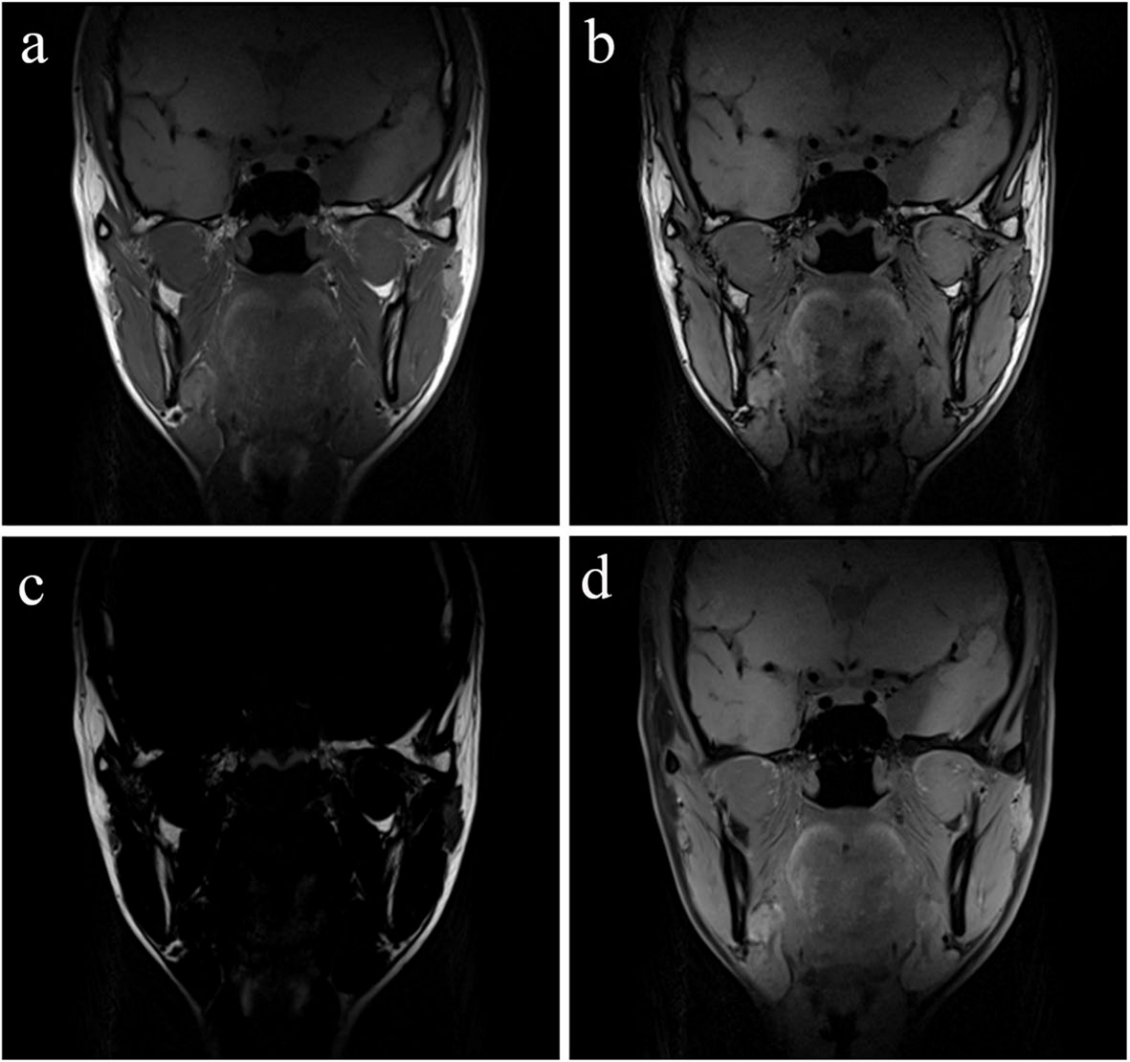
Four phases of images obtained by coronal T1-weighted Dixon sequence. (**A**) In-phase image. (**B**) Opposed-phase image. (**C**) Fat image. (**D**) Water image

The four phases of images are shown in Fig. 2. In the open-mouth position, oblique sagittal and coronal proton density-weighted sequence with fat saturation were obtained. The detailed parameters are shown in Table 1.

**Table 1:** Parameters of the MRI sequences

Parameters	Axial T2WI	Oblique Sagittal PDWI-FS	Oblique Sagittal T1WI	Oblique Coronal PDWI-FS	Coronal Dixon T1WI
TR (ms)	3000	3400	600	2500	739
TE (ms)	125	26	11	24	9.9 (In-phase)/Automatically calculated the Opposed-phase TE
Acquisition matrix	384 × 384	320 × 224	320 × 195	256 × 256	320 × 240
Reconstructed voxel size (mm)	0.57 × 0.57 × 3.00	0.31 × 0.31 × 2.00	0.31 × 0.31 × 2.00	0.39 × 0.39 × 2.00	0.69 × 0.69 × 3.00
Field of view (mm)	220 × 220	100 × 100	81.3 × 100	100 × 100	206.3 × 220
Slice Thickness (mm)	3	2	2	2	3
Number of averages	1	2	2	2	1
Flip angle (°)	90	150	120	150	128
Acquisition Time (s)	36	202	119	204	152

Note. *PDWI-FS*, Proton density-weighted with fat saturation, T2WI, T2-weighted image, *T1WI-T1* weighted image, *TR*, Repetition time, *TE*, Echo

### Data analysis

Firstly, observer A (S.W., with 6 years of experience in dentistry and 1 year of experience in oral radiology) and observer B (Y.C., with 2 years of experience in oral radiology) received 3–5 h of one-on-one training from observer C (D.J.S., with 8 years of experience in oral radiology). The training content included: delineation of the regions of interest (ROI) of LPM; measurement of the length, width and thickness of LPM. The detailed evaluation criteria are described in “Fat fraction of LPM” section and “Morphological features of LPM” section of Methods. Then, observer A and observer B performed standardization session with consensual evaluation of 10 randomly selected cases to improve the reliability 1 week later, and the study was then formally started. In this study, the TMJ disk position was all evaluated by observer A blinded to the MRI result and clinical data. 114 joints were divided into three groups according to the disk position: NP group, ADDWR group and ADDWOR group. In order to match sex ratio among the three groups, 2 female patients with bilateral ADDWR joints and 2 female patients with bilateral ADDWOR joints were randomly excluded, and a total of 106 joints were eventually included in the evaluation (Fig. 2). The fat fraction, morphological and texture features extracted from in-phase image of LPM of the 106 joints were evaluated by observer A and observer B blinded to patient data. The results of the observer A were used for statistical analysis.

### Fat fraction of LPM

**Fig. 3:**
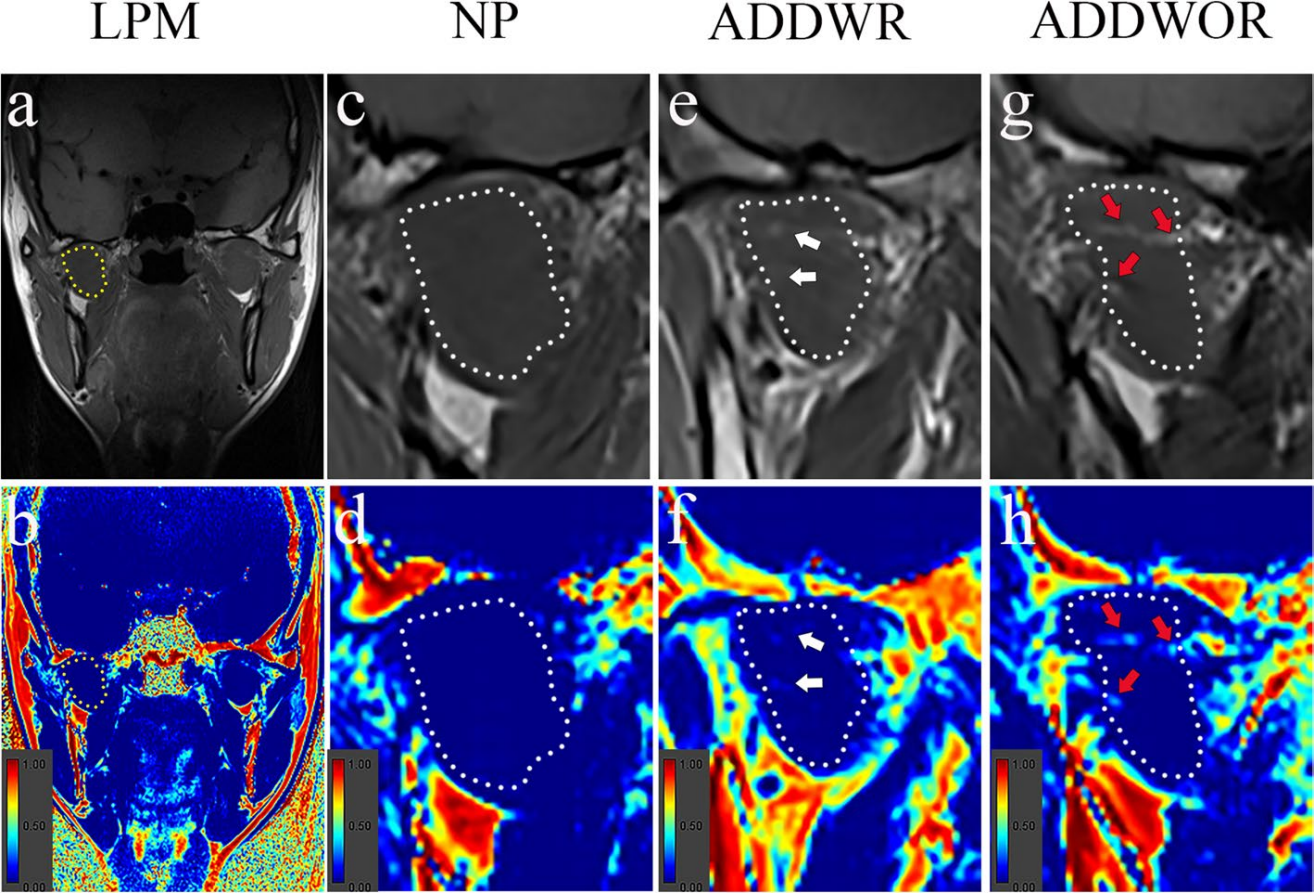
Fat fraction of lateral pterygoid muscle (LPM) in normal position disk (NP) group, anterior disk displacement with reduction (ADDWR) group and anterior disk displacement without reduction (ADDWOR) group. Yellow dotted line shows the ROI in in-phase image (**a**) and fat fraction image (**b**). The LPM of NP group shows no obvious fatty infiltration in in-phase image (**c**) and fat fraction image (**d**). (**e**, **f**) Slight fatty infiltration (white arrow headed) showed in the white dotted line in the ADDWR group, (**g**, **h**) while obvious fatty infiltration (red arrow headed) showed in the ADDWOR group

The fat fraction image was calculated from the signal intensity of fat image and water image by ImageJ (1.80 version; NIH, Bethesda, MD, USA) with the following equation [25]:$\mathrm{Fat fraction image }\left(\mathrm{\%}\right)=\frac{{\mathrm{Signal Intensity }}_{\mathrm{Fat image}}}{{\mathrm{Signal Intensity }}_{\mathrm{Fat image}}+{\mathrm{Signal Intensity}}_{\mathrm{ Water image}}}\times 100$. The detailed steps of this process were performed as follows: Firstly, the signal intensity of each voxel in the fat image and water image were added to produce a superposition image. Then, the signal intensity of each voxel of fat image was divided by the signal intensity of each voxel of the superposition image to produce the fat fraction image. In addition, as the low to intermediate signal muscle contrasts well with the high signal peripheral fat of the in-phase image. Thus, the in-phase image was chosen to draw the ROIs, which offers more distinct boundary of the LPM than the other three phases of images. Importing the in-phase image into ImageJ, then the ROIs were manually delineated along the boundary of LPM while avoiding the fascia of LPM **(**Fig. 3a**)**. Afterwards, the ROIs of in-phase image were copied to the fat fraction image to calculate the fat fraction **(**Fig. 3b**)**. The largest cross-sectional area layer and two adjacent layers of LPM were selected for the measurement. The mean value of these three layers was used as final result.

### Morphological features of LPM

**Fig. 4:**
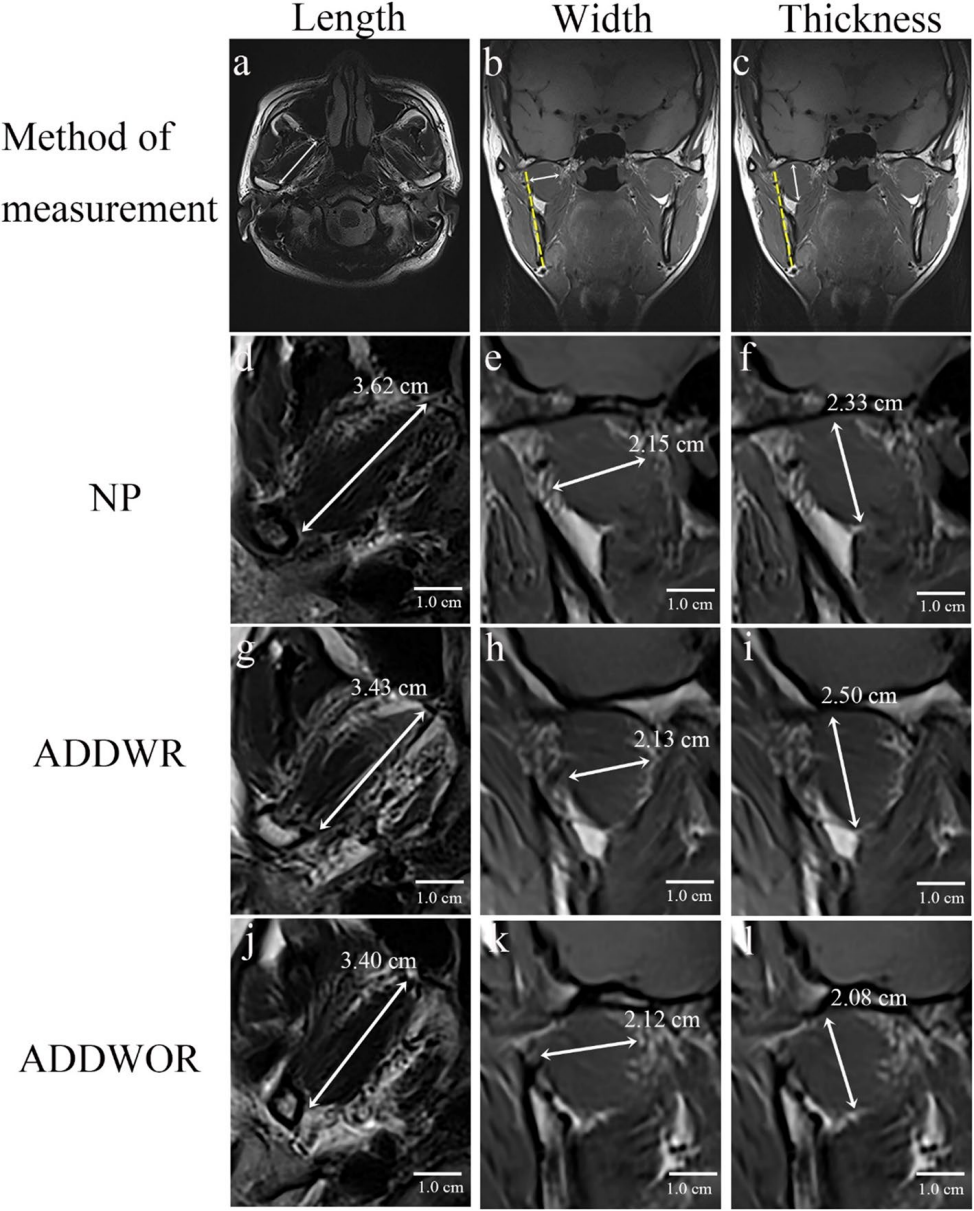
The morphological features of lateral pterygoid muscle (LPM) in normal position disk (NP) group, anterior disk displacement with reduction (ADDWR) group and anterior disk displacement without reduction (ADDWOR) group. The measurement of length (**a**), width (**b**) and thickness (**c**) of LPM. As the anterior disk displacement became more and more severe, (**d**, **g**, **j**) the length and (**e**, **h**, **k**) the width of LPM showed a tendency of decrease but the thickness of LPM (**f**, **i**, **l**) increased firstly and then decreased

The morphological change of LPM was evaluated from length, width and thickness of LPM. RadiAnt DICOM viewer (2020.2.2 version, Medixant, Poznań, Poland) was used to perform the measurement as follows. Length: Maximum length of LPM parallel to the long axis of LPM on the axial dimension (Fig. 4a**)**. Width: The longest diameter of LPM perpendicular to the long axis of mandibular ramus on the coronal dimension (Fig. 4b**)**. Thickness: The longest diameter of LPM parallel to the long axis of mandibular ramus on the coronal dimension (Fig. 4c**)**. In this retrospective study, we had only scanned the axial T2-weighted sequence without the coronal T2-weighted sequence, and the coronal T1-weighted Dixon sequence without scanning the axial T1-weighted Dixon sequence. Thus, the axial T2-weighted sequence was chosen to measure the length of LPM, and the in-phase image (which offers more distinct boundary of the LPM than the other three phases of images) of coronal T1-weighted Dixon sequence was chosen to measure the width and thickness of LPM.

### Texture features of LPM

Texture analysis was performed on in-phase image, which was imported into ImageJ and converted to 8-bit image. Selecting the previous layer and ROIs used to calculate fat fraction to analyze the texture features by Gray-level cooccurrence matrix Texture plug (http://imagej.nih.gov/ plugins/texture.html). “Size of the step in pixels” was set to 1 and “direction of the step” was set to 0 degree. Five texture features were obtained: Angular second moment (ASM), Contrast (CON), Correlation (COR), Inverse different moment (IDM) and Entropy (ENT).

### Statistical analysis

Statistical analysis was performed using Graph Pad Prism (Version 9.0, La Jolla, CA, USA) and IBM SPSS (Version 26.0, Armonk, NY, USA). Shapiro–Wilk test and q-q plot were used to assess the normal distribution of data. The age and sex ratio among groups were compared using Kruskal–Wallis test and chi-square test respectively. One-way ANOVA with Bonferroni correction was performed to compare the fat fraction, width, thickness and ENT among groups. Welch’s ANOVA was performed to compare the length and IDM among groups. Kruskal–Wallis test was performed to compare ASM, CON and COR among groups. Relationships between fat fraction and age, and texture features were evaluated by Spearman correlation coefficient (ρ). Relationships between fat fraction and morphological features were evaluated by Pearson correlation coefficient (r). The r or ρ indicated correlations as follow: less than 0.3, weak; 0.3–0.7, moderate; above 0.7, strong. Intra-class correlation coefficient (ICC) was performed to evaluate the inter-observer and intra-observer agreement. Two tailed *P* value less than 0.05 was considered as statistically significant.

## Results

### Patients and joints characteristics

Characteristics of patients and joints are presented in Table 2.

**Table 2:** Characteristics of patients and joints

Characteristics	Value
No. of NP	30
Age of NP (years)	26 (21,37) ^*^
No. of female joints / No. of male joints in NP	18/12
No. of ADDWR	27
Age of ADDWR (years)	25 (23,28) ^*^
No. of female joints / No. of male joints in ADDWR	21/6
No. of ADDWOR	49
Age of ADDWOR (years)	25 (20,44) ^*^
No. of female joints / No. of male joints in ADDWOR	41/8

Note. -Unless otherwise specified, the data are number of patients and joints, and percentages in parentheses. *NP*, Normal position disk, *ADDWR*, Anterior disk displacement with reduction, *ADDWOR* Anterior disk displacement without reduction

^*^Data are median age, with interquartile range in parentheses

A total of 106 joints (median, 25 years; interquartile range, 21–31; 80 female joints [75%]) from 53 patients (median, 25 years; interquartile range, 21–32; 40 female patients [75%]) were collected. There was no significant difference in age and sex ratio among NP group, ADDWR group and ADDOWR group (*P* = 0.666 and 0.057 respectively).

### Fat fraction of LPM

**Fig. 5:**
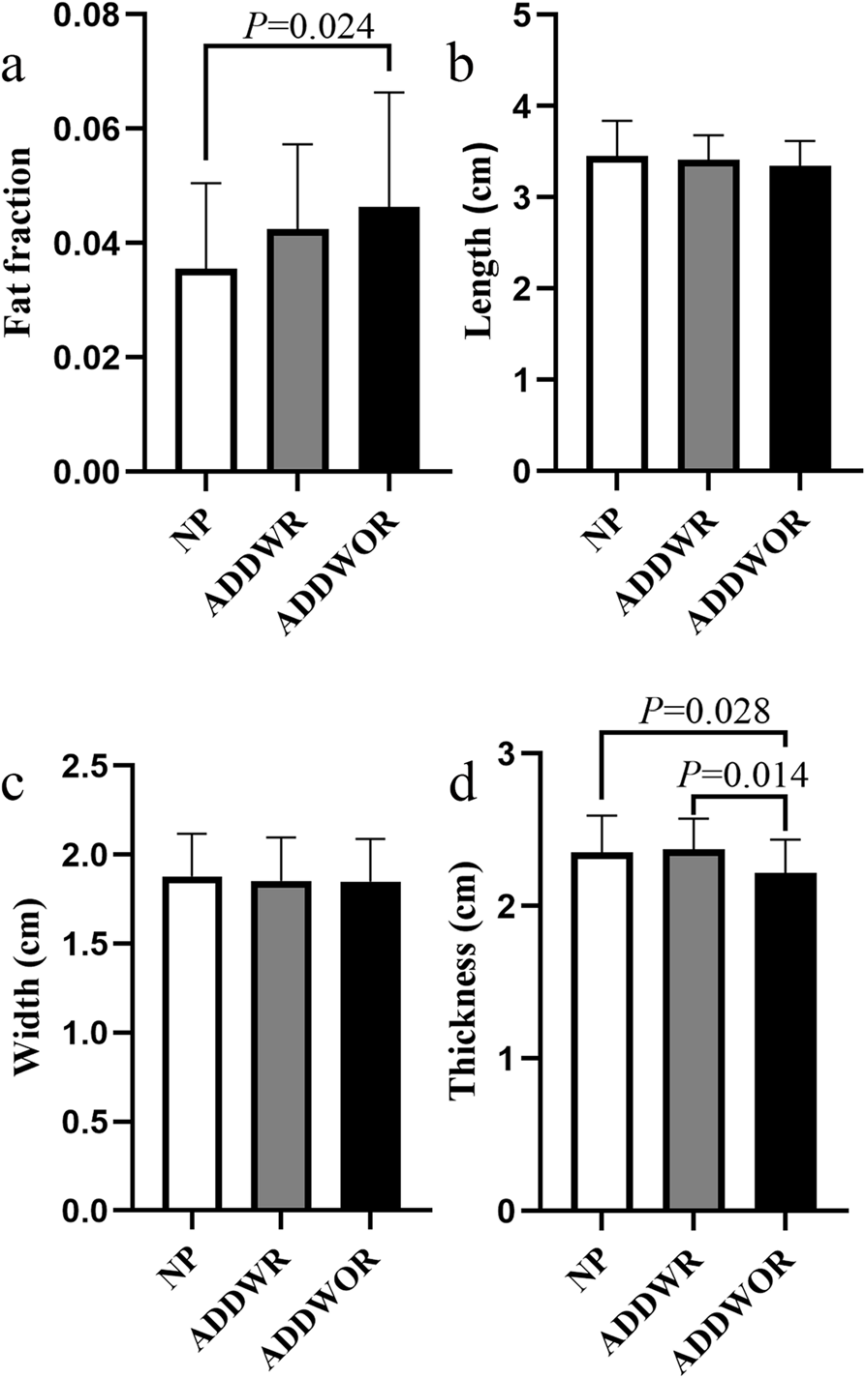
The Fat fraction and morphological features of lateral pterygoid muscle in normal position disk (NP) group, anterior disk displacement with reduction (ADDWR) group and anterior disk displacement without reduction (ADDWOR) group. (**a**) There is a significant difference of fat fraction between NP group and ADDWOR group. (**b**, **c**) There is no significant difference of length and width among three groups. (**d**) There is a significant difference of thickness between NP group and ADDWOR group, ADDWR group and ADDWOR group

**Fig. 6:**
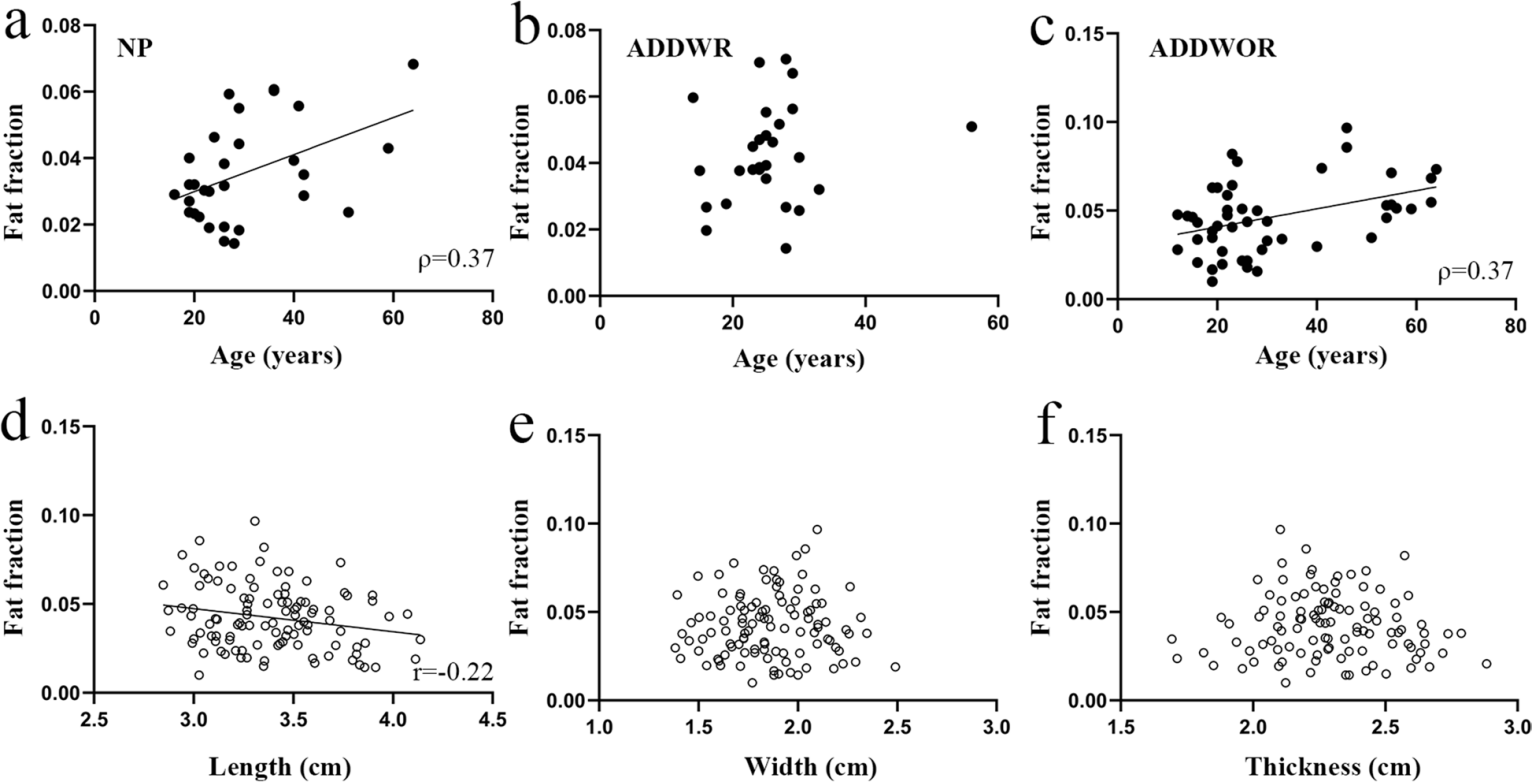
Relationship between fat fraction and age, and morphological features of lateral pterygoid muscle. NP, Normal position disk, ADDWOR, Anterior disk displacement without reduction. (**a**, **c**) There is a moderate positive correlation between fat fraction and age in NP group and ADDWOR group, (**b**) while no significant correlation is found in ADDWR group. (**d**) There is a weak negative correlation between fat fraction and length, (**e**, **f**) while fat fraction is not found significantly correlated with width and thickness

The results of fat fraction of LPM among three groups are shown in Table 3 and Fig. 5a. Figure 3**(**c-h**)** depicts the example of the fatty infiltration of LPM within three groups. The ADDWOR group showed significantly higher fat fraction than NP group (4.63 ± 2.01% vs 3.65 ± 1.5%; *P* = 0.024). The correlation analysis of relationship between fat fraction and age are shown in Table 4 and Fig. 6 (a-c). The ICC of Inter-observer agreement and Intra-observer agreement of fat fraction were 0.83 and 0.91 respectively (Table 5).

**Table 3:** Fat fraction, morphological and texture features of LPM

Variable	NP	ADDWR	ADDWOR	*P* Value
Fat fraction (%)	3.65 ± 1.50	4.25 ± 1.48	4.63 ± 2.01	0.032^*^
Morphological features				
Length (cm)	3.45 ± 0.39	3.42 ± 0.27	3.35 ± 0.27	0.367^‡^
Width (cm)	1.88 ± 0.24	1.85 ± 0.24	1.85 ± 0.24	0.868^*^
Thickness (cm)	2.35 ± 0.24	2.37 ± 0.20	2.22 ± 0.22	0.005^*^
Texture features				
ASM (10^–3^)	2.33 (2.00–2.67)	2.00 (2.00–2.67)	2.00 (1.83–2.67)	0.361^†^
CON	374.4 (267.4–480.9)	321.9 (265.4–432.4)	337.5 (285.4–418.7)	0.819^†^
COR (10^–4^)	8.60 (6.89–9.97)	8.36 (5.67–13.33)	9.04 (7.72–12.16)	0.464^†^
IDM	0.18 ± 0.02	0.18 ± 0.03	0.18 ± 0.02	0.771^‡^
ENT	6.62 ± 0.21	6.62 ± 0.25	6.63 ± 0.21	0.977^*^

Note.—Data are represented by mean ± standard deviation or median (quartile range)

*LPM*, Lateral pterygoid muscle, *NP, *Normal position disk, *ADDWR*, Anterior disk displacement with reduction, *ADDWOR*, Anterior disk displacement without reduction, *ASM*, Angular second moment, *CON*, Contrast, *COR*, Correlation, *IDM*, Inverse different moment, *ENT*, Entropy

^*^ *P* value for the One-way ANOVA test among NP group, ADDWR group and ADDWOR group

^‡^ *P* value for the Welch’s ANOVA test among NP group, ADDWR group and ADDWOR group

^†^* P* value for the Kruskal–Wallis test among NP group, ADDWR group and ADDWOR group

**Table 4:** Correlation analysis of fat fraction with age, morphological and texture features of LPM

Variable	*ρ*/r Value	*P* Value
Age		
Age (NP)	0.37^*^	0.041
Age (ADDR)	0.16^*^	0.427
Age (ADDWOR)	0.37^*^	0.009
Morphological features		
Length (cm)	-0.22^†^	0.026
Width (cm)	0.02^†^	0.848
Thickness (cm)	-0.06^†^	0.567
Texture features		
ASM	-0.32^*^	< 0.001
CON	0.17^*^	0.090
COR	-0.28^*^	0.003
IDM	-0.27^*^	0.005
ENT	0.31^*^	0.001

Note. *LPM*, Lateral pterygoid muscle, *NP*, Normal position disk, *ADDWR*, Anterior disk displacement with reduction, *ADDWOR*,  Anterior disk displacement without reduction, ASM = Angular second moment, *CON,* Contrast, *COR*,  Correlation, *IDM*, Inverse different moment, *ENT*, Entropy

^a^*ρ* value of Spearman correlation analysis

^†^r value of Pearson correlation analysis

**Table 5:** Intra-class correlation coefficient of fat fraction, morphological and texture features of LPM

Variable	Inter-observer agreement	Intra-observer agreement
Fat fraction (%)	0.83 (0.57–0.91)	0.91 (0.84–0.95)
Morphological features		
Length (cm)	0.90 (0.84–0.93)	0.86 (0.79–0.91)
Width (cm)	0.80 (0.71–0.86)	0.81 (0.73-.087)
Thickness (cm)	0.89 (0.85–0.93)	0.92 (0.88–0.94)
Texture features		
ASM	0.87 (0.80–0.91)	0.85 (0.79–0.90)
CON	0.96 (0.94–0.97)	0.97 (0.95–0.98)
COR	0.86 (0.80–0.90)	0.86 (0.81–0.91)
IDM	0.95 (0.89–0.97)	0.94 (0.91–0.96)
ENT	0.87 (0.77–0.92)	0.88 (0.82–0.92)

Note. Data are intraclass correlation coefficient, and 95% confidence intervals are shown in parentheses. *LPM*, Lateral pterygoid muscle, *ASM*, Angular second moment, *CON*, Contrast, *COR*, Correlation, *IDM*, Inverse different moment, *ENT*, Entropy

### Morphological features of LPM

The results of morphological features of LPM among three groups are shown in Table 3 and Fig. 5 (b-d). Figure 4 (d-l) depicts the example of the morphological features of LPM within three groups. The thickness of ADDWOR group was significantly decreased compared with NP group (2.22 ± 0.22 cm vs 2.35 ± 0.24 cm, *P* = 0.028) and ADDWR group (2.22 ± 0.22 cm vs 2.37 ± 0.20 cm, *P* = 0.014), respectively. The correlation analysis of relationship between fat fraction and morphological features are shown in Table 4 and Fig. 6 (d-f). All the ICC values of morphological features were ranged from 0.80 to 0.92 (Table 5).

#### Texture features of LPM

**Fig. 7:**
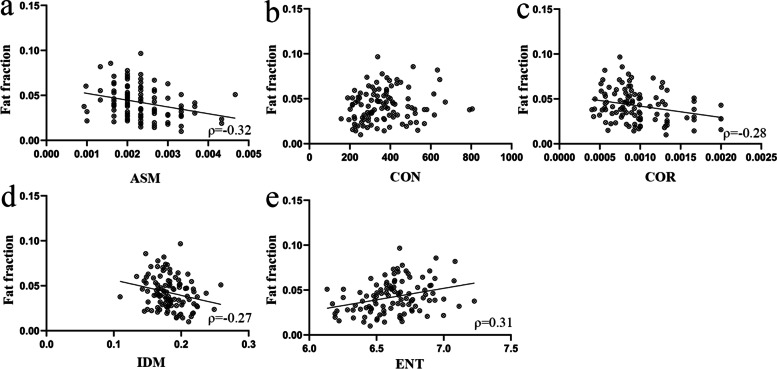
Relationship between fat fraction and texture features extracted from in-phase image of T1-weighted Dixon sequence of lateral pterygoid muscle. ASM,  Angular second moment, CON , Contrast, COR , Correlation, IDM, Inverse different moment, ENT, Entropy. (**a**) Fat fraction was moderately negatively correlated with ASM, (**c**, **d**) and was weakly negatively correlated with COR and IDM. (**e**) Fat fraction was moderately positively correlated with ENT, (**b**) but not significantly correlated with CON

The results of texture analysis of in-phase image of LPM among three groups are shown in Table 3. No significant difference in all the texture features was found among groups. The correlation analysis of relationship between fat fraction and texture features extracted from in-phase image are shown in Table 4 and Fig. 7. All the ICC values of texture features extracted from in-phase image of LPM were ranged from 0.85 to 0.97 (Table 5).

## Discussion

Fatty infiltration of LPM is associated with ADD [2]. Dixon technique can quantify the fat fraction in tissue and has already been studied in head imaging. However, no quantitative study on the fat fraction of LPM has been reported. In this research, our results showed the fat fraction of LPM in NP group was 3.65%. Studies have found that the fat fraction of skeletal muscle (thigh or calf) in healthy people was ranged from 2 to 5% [26, 27], and our fat fraction value of NP group just belonged to the range that described above. Recently, keene et al. [17] reported on the temporal muscle, which is also a masticatory muscle, with a fat fraction of 9.3%, slightly higher than the fat fraction of LPM (ranged from 3.65% to 4.63%) we observed in this study. The closer to the orbital fat, the more noise bias is likely to occur in the imaging of nearby muscles, and resulting in an overestimation of fat fraction. Thus, the slightly lower fat fraction of LPM we observed might be due to the fact that LPM is farther from orbital fat than temporal muscle [17]. Of course, different sequence parameters such as repetition time and the number of echoes might also influence the assessment of fat fraction [25].

In our study, subtle difference in the fat fraction of LPM between NP group and ADDWOR group were detected using the Dixon technique (NP group: 3.65%; ADDWOR group: 4.63%). This result means the fat fraction of LPM tended to increase linearly with the aggravation of ADD, which also indicated that Dixon technique is sensitive to subtle fatty infiltration of LPM. This might be because the double-echo or multi-echo Dixon technique based on water-fat separation method can simultaneously collect images with different phases [25], thereby reducing the scanning time and thus circumvent the magnetic field inhomogeneity induced by rich tissue-air interfaces around the LPM [15]. Therefore, the fat fraction of LPM quantified by Dixon technique might be a sensitive marker to judge whether the patient with ADD had undergone minor progression or not.

Nagamsom et al. [4] performed a quantitative evaluation on intravoxel incoherent motion MRI and reported blood perfusion fraction of LPM in ADDWOR was significantly higher than that of NP (NP group: 0.33 × 10^−3^mm^2^s^−1^; ADDWOR group: 0.40 × 10^−3^mm^2^s^−1^), implying increased inflammation of LPM with the aggravation of ADD. Liu et al. [6] used diffusion tensor imaging MRI to evaluate the apparent diffusion coefficient and eigenvalues of LPM in patients with TMD, and found that the above parameters increased nearly linearly with the severity of ADD (eg: apparent diffusion coefficient in NP group: 1.530 × 10^−3^mm^2^s^−1^;apparent diffusion coefficient in ADDWOR group: 1.632 × 10^−3^mm^2^s^−1^), suggesting the microstructure of LPM has undergone pathological changes. Our results also showed the fat fraction of LPM in ADDWOR group was significantly higher than that in NP group. This trend of pathological status of LPM to gradually worsened with the aggravation of ADD is consistent with our study. A recent study retrospectively collected T1-weighted turbo spin-echo images from 77 TMD patients and observed increased T1 signal intensity of LPM in ADDWR and ADDWOR compared to NP [5], suggesting the fatty infiltration of LPM was severer in the ADDWOR group, which was also similar to our findings. However, the T1 signal intensity used in their study could not completely represent the fat content, as the T1 signal intensity is also affected by other factors.

The higher incidence of fatty infiltration of LPM in ADDWOR might be due to the following reason: muscle disuse can lead to loss of muscle fibers and accumulation of lipid or fat [7]. In ADDWOR, the disk cannot return to its normal position, and the LPM that control the stability of disk cannot function properly and tend to be disused, which might lead to more severe fatty infiltration of LPM in ADDWOR than that in NP or ADDWR. On the other hand, the accumulation of lipid or fat in muscle is currently known to be related with insulin insensitivity and inflammation, which impair the muscle function [7]. Since the LPM plays an important role in stabilizing the TMJ disk, thus, the loss of disk control caused by weakened function of fatty infiltrated LPM might also promote the progression of NP or ADDWR to ADDWOR. In addition, it was observed in this study that the bands of fatty infiltration (white and red arrows headed in Fig. 3e, f, g, h) divided the LPM into smaller upper part and bigger lower part, which was consistent with the morphological characteristics of LPM with smaller superior head and bigger inferior head [1, 2]. Fatty infiltration in muscle was often present along the fascia [22], thus, it can be speculated that the increased in fatty infiltration of LPM might be more pronounced in the fascia area between superior head and inferior head.

It is well known that the extent of fatty infiltration of skeletal muscle also increases with aging even in healthy human and animal model [28, 29]. Therefore, it is understandable that a moderate correlation was found between fat fraction and age in NP group. However, it is interesting that moderate correlation was also found between fat fraction and age in ADDWOR group. The reason for this might be that the disk in ADDWOR group is always in front of the condyle, maintaining a special relative stability [2]. As a result, the activity of LPM, which maintains the stability of disk, is also relatively stable. This special stable state allows the fat fraction of LPM established on a new equilibrium relationship. It has been reported that the LPM might be normal or hypertrophic in ADDWR, while often undergone atrophy in ADDWOR [1, 30]. Our study also observed that thickness of LPM increased in ADDWR while decreased in ADDWOR, which seem to be consistent with their results. Shor-term excessive overloading of skeletal muscle leads to hypertrophy, while atrophy is considered secondary to long-term excessive overloading [2]. Hence, it can be speculated that the increase in thickness during ADDWR might be related to the hypertrophy caused by short-term excessive overloading.

In ADD, the proportion of atrophy of the superior head was much higher than that of the inferior head [1, 2]. The reason for the higher proportion of superior head atrophy than that of inferior head might be due to the superior head more closely linked to the disk, leading to the more reduction of the muscle function after ADD, which in turn could cause the superior head to be more prone to be atrophy than the inferior head [1]. In our study, each measurement of thickness included the superior head, but the upper head was not always included in the measurement of length and width. Understandably, significant change in the length and width of LPM among groups was not observed. This study also found the length was negatively correlated with fat fraction. Keller et al. [31] observed negative correlation between fat fraction and fiber track length of dystrophic skeletal muscle. As the length of LPM was defined as the maximum parallel to the LPM, so it can be inferred that the length of LPM fibers might become shorter with the fatty infiltration.

Texture analysis can be used to convert medical images into quantitative descriptors of specific tissue [19]. Gray-level cooccurrence matrix is a second-order texture analysis method, which can characterize the spatial distribution of pixels from the grayscale information by detecting the relationship between two points at different distances and directions [18, 32]. It has been widely applied in clinic practice such as head and neck cancer [19], myocardial infarction [18], Duchenne muscular dystrophy [23] and dynapenia [20], but its research on TMD is still at a preliminary stage. Recently, Liu et al. [32] used gray-level cooccurrence matrix to analyze the texture features of LPM in 29 patients with TMD on T2-weighted image, and observed significant decrease of ENT in ADDWOR group compared to ADDWR group. It has been reported that the in-phase image based on Dixon technique could provide the highest numbers of reproduceable texture features [16], thus, the in-phase image was chosen as the representative image of texture analysis in this study. However, no significant differences in texture features of LPM were detected among groups in our study. The function of LPM is heterogeneous in different segments [33]. Our research was to analyze the middle segment of LPM on the coronal plane, while Liu’s study was to analyze the layer of maximal area on the axis plane. The ROI selected in different segments of LPM might be the reason for our inconsistent results. Studies have observed that there was a positive correlation between fat content and ENT in skeletal muscle [34, 35], which is consistent with our study. Heterogeneous image would have smaller ASM, COR, IDM, and greater CON and ENT [36]. Interestingly, our results of texture analysis of in-phase image also showed that as fat fraction increased, ASM, COR, IDM became smaller and ENT became greater. This suggested that fatty infiltration is associated with increased intramuscular heterogeneity of LPM. The reason for this might be related to the coarseness of the muscle texture after fatty infiltration, which led to the irregular and uneven grayscale information of the image [23].

There had several limitations in our research. First, the sample size in this retrospective study was small and only in a single institution. In addition, when grouping the samples, the TMD group and the healthy control group were not distinguished, nor did distinguish whether the subject had osteoarthritis or not. These factors might limit the power of our study. Second, even the age and sex ratio were controlled among groups, other confounders that might influence the fatty infiltration of LPM were not controlled. For example: body mass index, duration time of ADD, and unilateral chewing habit, etc. Third, other texture analysis [21] such as histogram analysis, run-length matrix analysis are also needed to be involved in the future study. Forth, the two-dimensional delineation of LPM in this study might miss the heterogeneity of the entire muscle compared with the three-dimensional delineation [24]. Future study needs to design three-dimensional sequence to comprehensively analyze the heterogeneity of LPM. Fifth, the LPM was analyzed as a whole without separating the superior head and inferior head from each other. In this study, the two heads of LPM can only be distinguished on very few levels when delineating the ROI, while lopes et al. [37] also held the same opinion that it is very difficult to define the boundary between superior head and inferior head. Therefore, in order to ensure the accuracy of measurement, the LPM was still considered as a single muscle.

## Conclusions

Fatty infiltration of LPM in ADDWOR group was severer than it in NP group. The severer the fatty infiltration of LPM, the more atrophic and increased intramuscular heterogeneity of the muscle. Fat fraction of LPM evaluated by Dixon sequence could be a potential marker of ADD disease process.

## Data Availability

The datasets used and analyzed in this study are available from the corresponding author on reasonable request.

## Abbreviations

LPM: Lateral pterygoid muscle
ADD: Anterior disk displacement
TMJ: Temporomandibular joint
NP: Normal position disk
ADDWR: Anterior disk displacement with reduction
ADDWOR: Anterior disk displacement without reduction
ICC: Intra-class correlation coefficient
TMD: Temporomandibular joint disorders
ROI: Regions of interest
ASM: Angular second moment
CON: Contrast
COR: Correlation
IDM: Inverse different moment
ENT: Entropy
